# Nearness to God: Danish Muslims and *Taqwa*-infused faith frames

**DOI:** 10.1007/s11562-022-00498-2

**Published:** 2022-10-27

**Authors:** Maria Lindebæk Lyngsøe, Simon Stjernholm

**Affiliations:** Department for Cross-Cultural and Regional Studies, University of Copenhagen, Copenhagen, Denmark

**Keywords:** Piety, *Taqwa*, Prayer, Instruction, Faith frame, Denmark

## Abstract

This article advocates for an increased attention to how piously striving Muslims learn about, cultivate, and experience nearness to God. The empirical material is taken from our current research on Danish Muslims’ engagement with Islamic teaching and learning. We examine examples of oral teachings that instruct the audience to be constantly aware of God and address him directly in prayer, examples of how an awareness of God is cultivated and practiced in everyday life, and individual narratives of experiences of being close to or helped by God. With inspiration from the anthropology of Christianity as well as Islam, we propose an analytical model for understanding the process whereby Muslim efforts to draw near to God can ‘work’. Three interrelated dynamics are crucial to this process, and we identify each through our reading of existing scholarship. The dynamics at play are, respectively, a *taqwa*-infused faith frame, its related semiotic ideology, and a relationship of experienced reciprocal agency with God.

## Introduction

I feel like I live in the real world, whereas the world you live in with non-religious people is the non-real world. […] It can be hard to act among others, and I feel that it sets certain limitations as to whom I talk to about what. If it is non-religious people who just don’t understand that mode of being, I hold back many things because I know they would not be able to understand. (Interview with Louise, May 2019)

This was uttered by Louise, a Danish university student and convert to Islam in her early 30s, when she reflected upon the fact that her mode of being in the world set her apart from most Danes. Louise made clear that being religious, for her, had to do with a certain ontology—living in a world that is fundamentally different from a world without (the possibility of) God’s existence, presence and nearness. To some degree she thought about, expected and experienced things differently from non-religious people around her, and this affected her mode of being in the world.

In our research on pious Danish Muslims’ engagement with Islamic teaching, preaching, learning, and living a religious life, we have found that individuals’ nearness to God as an ideal and as experience is an important concern. Yet existing studies on Muslims in Europe seldom focus on pious interiority or experiences of an intimate relationship to God (cf. Fadil, 2019). Rather, issues concerning politics, identification, media, secularity, representation, and organisation are often highlighted (to give but a few recent examples concerning the Scandinavian countries specifically, see e.g. Bangstad, 2014; Jensen, 2019; Lundby, 2018; Otterbeck, 2010; Thurfjell & Willander, 2021; see also Rytter, 2019a). While these topics are important and address central questions in the study of contemporary Islam, in this article we advocate for an increased attention to how piously striving Muslims learn about, cultivate, and experience nearness to God. The article takes inspiration from existing anthropological studies on Muslims, mostly in non-European geographical contexts, as well as studies within the history and anthropology of Christianity. Moreover, we integrate the classical Islamic concept of *taqwa* with these academic perspectives in our analysis of Muslim nearness to God.

The empirical material presented in this article is taken from our current research on Danish Muslims’ engagement with Islamic teaching and learning. We examine examples of oral teachings that instruct the audience to be constantly aware of God and address him directly in prayer, examples of how an awareness of and nearness to God is cultivated, felt, and practiced in everyday life, and individual narratives of experiences of being close to or helped by God. Through our analyses, we find that striving to establish nearness to God appears to be fundamental to pious Danish Muslims—both individuals and groups. Moreover, awareness and experiences of God’s nearness and presence might both result from and lead to deepened engagement with Islamic knowledge. In the following section, we introduce our conceptual framework and propose an analytical model for understanding the process whereby Muslim efforts to draw near to God and sense his nearness can ‘work’.

## Nearness to God: conceptual and analytical framework

A conceptual combination of recent academic research on pious Muslims and Christians with selected concepts concerning piety developed in classical Islamic tradition can prove fruitful for analysing contemporary Muslims’ endeavours to achieve nearness to God. A number of recent works—especially within anthropology—have considered how and to what extent it makes sense to include notions of divine and other non-human entities as active and agentive presences in analyses of pious religiosity (see e.g. Bialecki, 2014, 2016; Furani & Robbins, 2021; Luhrmann, 2007, 2012, 2020; Mittermaier, 2011, 2012, 2019; Orsi, 2012, 2016; Robbins, 2001, 2017, 2019; Schielke, 2019). It has been suggested that religious subjects might be best understood as implicated and engaged in ‘webs of relationships’ that include non-human actors who are experienced as being able to ‘act upon’, or significantly impact, human subjects and the world humans inhabit (Mittermaier, 2012: 249, 252). This alleged impact can be indexed by a number of signs, which are perceived and interpreted by humans through processes of semiosis. Semiotic ideologies, a concept developed by Webb Keane, are ‘assumptions about what signs are, what functions signs do or do not serve, and what consequences they might or might not produce’ (Keane, 2018: 65; see also Keane, 2003, 2007). The relevance, meaning, and effect ascribed to a sign—for example, a certain event or utterance—will vary according to the particular semiotic ideology through which a religious subject encounters and interprets the world. This has consequences for how individuals can establish relationships to the divine—as well as what kinds of relationships that are seen as possible.

Tanya M. Luhrmann’s (2012, 2020) work on the prayer practices of groups of Christians in the USA and elsewhere addresses precisely the nature and possibility of such relationships. She uses the concept ‘faith frame’, by which she means ‘a mode of thinking in which gods and spirits really matter’, in contrast to a ‘set of expectations about an everyday world’ in which that is not the case (2020: 21). Luhrmann herself encourages comparative use of the faith frame concept across religious traditions, as she briefly notes that there are notions for this type of awareness in Islam (she mentions the term *taqwa*, which we elaborate on below), the Hebrew Bible, the New Testament, and the Bhagavad Gita (2020: 22). Taking a concept that has been developed in the context of one religious tradition and using it in a different religious context always comes with potential pitfalls. Although Luhrmann’s analyses are highly oriented toward practices, the concept of ‘faith frame’ clearly prioritises ‘faith’ as an inner form of religiosity, which, as many have pointed out, does not play an equally central role in all religious traditions (cf. Asad, 1993; Keane, 2007). Nonetheless, we find that Luhrmann’s concept of faith frame captures the relationship between inner processes and bodily practices and offers fruitful ways of engaging with Islamic forms of piety. There are of course differences regarding what ‘faith’ means to Christians and Muslims, yet Islamic traditions by no means lack concepts and practices that relate to inner forms of piety and connections to the divine.

Ideas and ideals regarding how to develop and experience nearness to God has a long and complex history within Islamic spiritual and theological traditions. A concept of particular concern is *taqwa*, which can be translated as, for example, ‘godliness’, ‘devoutness’, ‘god-fearing piety’, ‘god-consciousness’, or ‘devotion’ (Lewisohn, 2012).^1^ Variants of the term are highly present in the Qurʾan (Ohlander, 2005) as well as in hadith literature, and have been elaborated on as a quality of interiorised faith by a number of significant Islamic scholars (Lewisohn, 2012). Erik S. Ohlander’s (2005: 149) analysis of semantic shifts and thematic contexts related to *taqwa* in the Qurʾan shows that in suras ascribed to the Medinan period, the ‘fear’ it implies is ‘no longer simply a psychological state brought on by eschatological warnings but is rather cast as a moral virtue to be cultivated’. *Taqwa* is thus presented as piety more generally, ‘in the sense of an active and conscious devotion and deference to God and His will’. Ohlander also describes *taqwa* as a ‘religious conscience’ and connects it to ‘spiritual matters and the relationship between the believer and his Lord’ (Ohlander, 2005: 150). The concept of *taqwa* clearly has a lot to do with the type of faith frame that can be expected to encourage and produce experiences of nearness to God and his work in the world. It therefore makes sense to talk of a *taqwa*-infused faith frame as an ideal in the Islamic settings we analyse here. We believe that the concept *taqwa*-infused faith frame, which combines Luhrmann’s terminology, a classical Islamic concept, and Mittermaier’s notion of being ‘acted upon’, has the potential to shed further light on important but underexposed aspects of contemporary Muslim piety.

Luhrmann examines the work that Christians in various locations put into staying within the faith frame ‘as much as they can, despite how easy it can be to get distracted or discouraged’ (2020: 22).^2^ She states that prayer can ‘work’ in the sense that it can make God feel more real and closer through practices of ‘kindling’. This makes it possible for the individual to engage in an active relationship with God in which s/he feels listened to, taken care of, and loved. To live within a certain faith frame, we suggest, can thus mean inhabiting the world through a particular semiotic ideology in which signs of God’s active presence are not only possible, but desirable and sought-after. While Luhrmann suggests that maintaining the realness of gods requires work from individual believers, this might be especially important in a context where one’s worldview is contested due to being in a minority position. This is in many ways the situation for Danish religious Muslims, who typically commit to a different ontology than the majority of Danes (Andersen et al., 2019; Poulsen et al., 2021: 23). This is reflected in the introductory quote from Louise, who distinguished between a ‘real’ world and a ‘non-real’ world. In such a minority context, individual Muslims like Louise are likely to be confronted with the particularity of their faith frame, which creates an accentuated need for a reflexive work to maintain one’s faith frame.

The faith frame, with its ideal ontological commitment and its related semiotic ideology, establishes normative knowledge about ‘right’ and ‘wrong’ as well as truths about what agents, words, and things can and cannot do. This knowledge is typically articulated through various types of normative religious discourse: preaching, teaching, edifying literature, and the like. Learning to think and act within a faith frame, and engaging in religious practices encouraged in such discourse, can be done with the desire to increase one’s personal piety. This kind of conscious pious self-cultivation can be central to individual projects aiming to achieve a certain pious habitus, as bodily actions and sensory engagements have been shown to shape ethical selves through training processes (Hirschkind, 2006; Mahmood, 2012). Yet as pointed out by Amira Mittermaier (2011, 2012, 2021; see also Rytter, 2016), we also do well to open up for the unanticipated, including alleged ‘divine interventions’ believed not to be caused by conscious pious self-cultivation, but rather by a will and agency experienced to be external to the pious subject. In order to better understand how pious Muslims might be entangled in webs of relationships with invisible beings, we need to be attentive to how such beings’ presence and agency are perceived and experienced (cf. Groeninck, 2017; Suhr, 2019). Therefore, we suggest that we allow for our analysis to capture not only the aspect of how individuals cultivate themselves in the attempt to come closer to God, but also ways in which they learn to interpret internal and external signs as experiences of God approaching or guiding them (Groeninck, 2017). This implies a certain reciprocity in the ways in which Danish Muslims talk about their relationship to God that we seek to capture in this analytical framework.

**Fig. 1:**
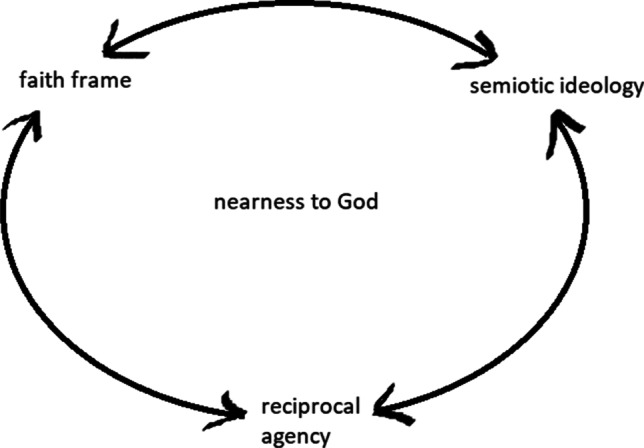
Dynamics of a sense of nearness to God

We have sought to illustrate the key points in this section in a simple model (Fig. 1), in which there is no necessary causal starting point but rather a mutually reinforcing circular flow. The model also indicates that disruptions that shatter a feeling of nearness can occur if one of its elements fails to ‘work’.

With this model in mind, and based on the above, we are now ready to pose more specific analytical questions: How do Danish Muslim preachers and teachers support *taqwa*-infused faith frames? How is nearness to God cultivated and supported through specific practices? What kinds of personal experiences of nearness to God do Danish Muslims report having? We will engage with these questions in the following, after having briefly introduced our methods and materials.

## Danish Muslims and nearness to God

The material we use in this article has been gathered since 2019 onwards in a research project focused on engagements with Islamic knowledge through teaching, preaching, and learning practices among Muslims in Denmark. We have collected data using qualitative interviews, participant observation, and by accessing various forms of publicly available digital media. The material gathered through interviews and observations has been pseudonymised, while the material gathered from public digital sources has not. In total, Lyngsøe has carried out 25 interviews with women engaged as teachers and students in Islamic educational activities. She has also participated in 54 formal and informal classes on Islamic topics, such as Qurʾan recitation, education aimed at converts to Islam, and various thematic lectures. These have primarily taken place in two mosques in the Greater Copenhagen area and—during the lockdown caused by the Covid-19 pandemic—on digital media platforms. Stjernholm, on the other hand, has listened to a large number of oral communications in Danish—such as lectures, sermons, Ramadan messages, ‘reminders’, podcast conversations, and radio broadcasts—created and disseminated by Muslim actors (some with a mosque affiliation, some independent). For the purposes of this article, we have selected examples from our respective pools of data that relate to our chosen topic: Muslim striving for nearness to God. We have compared and examined the examples together in light of our chosen analytical framework. In these analytical conversations, the three interrelated yet analytically distinct themes—learning about, cultivating, and experiencing nearness to God—gradually crystallised as key topics. We have therefore chosen to organise the analytical part of the article using this distinction.

### Learning that God is near

This section focuses on efforts at orally instructing Danish Muslims to live a life characterised by a *taqwa*-infused faith frame, meaning to be in a constant devotional awareness of and relationship to God. We also examine how individuals receive such instruction, as well as interlocutors’ reflections on how their mode of being in the world might differ from that of non-religious individuals, as expressed by Louise in the article’s introduction. There are multiple examples in our material of lectures and other oral communications where the speaker has emphasised the importance of being aware of their reciprocal relationship with God, that is, his capacity to always observe, listen to, and respond to individual believers’ actions and words. These examples illustrate how Danish Muslims are encouraged to inhabit a *taqwa*-infused faith frame, which, as indicated by Fig. 1, is meant to affect the semiotic ideology through which they interpret various signs, and thereby the types of experiences they are likely to have.

In a series of lectures circulated in January and February 2021, a couple of speakers representing the organisation MUNIDA (*Muslimsk Ungdom i Danmark*; Eng. *Muslim Youth in Denmark*) focused on these topics. In one of these lectures, the speaker took a certain hadith narrative as his point of departure for talking about *taqwa*, good deeds, and treating other people well.^3^ Regarding the first point, the speaker quoted Muhammad as saying ‘have *taqwa* of God wherever you are’.^4^ He then explained that *taqwa* means ‘being constantly […] aware that Allah *subhana wa taʿala* is the one who hears, he is the one who sees, he is the one who knows what you are thinking, what you are doing, what you are planning […], he is All-seeing and All-hearing’. In a further comment on the quoted hadith, the speaker emphasised that ‘wherever you are’ means that:

It is not enough to have *taqwa* [...] when you are with your friends, or when you are in the *masjid*, or when you are with family. No, you have *taqwa* wherever you are. Whether you are alone or in a congregation. Whether you are in your [...] home, the country you live in, or if you are travelling. Whether you are in school, at work, if you are ill, if you are well, if you are rich, if you are poor, if you are struggling, not struggling, happy, unhappy – wherever you are and in whatever situation you are in, you are conscious of Allah.

The speaker evidently wished to convey that there is no space, no time, and no state of affairs whatsoever where God does not see and hear you as well as know what you are thinking. The speaker further accentuated this point by saying that we sometimes do things when we are alone, when we know that no one can see or hear us, which we would be ashamed to do in front of others. In these moments, we forget that God always sees and hears us wherever we are. After having thus emphasised the importance of ubiquitous devoutness, the speaker went on to talk about the benefits of such a level of *taqwa*. He quoted the Qurʾan as saying, ‘the most noble and best among you is the one with the most *taqwa*’ (49:13), and that ‘for those who are Allah’s allies, those close to Allah, there is no fear or worry’ (10:62).^5^

In a lecture from February 2021, focus was on the importance of *duʿa* prayers in the individual Muslim’s life.^6^ The speaker quoted the Qurʾan as saying, ‘make *duʿa* to me and I will reply’ (40:60).^7^ He explained that this meant that ‘we are required to have this conviction […] that Allah *will* respond to our calls.’ The speaker then related two narratives to support his point. The first narrative was about the prophet Musa, who is said to have had mutual and direct communications with God. In the story, a childless woman approaches Musa in order to ask him to petition God to bless her with a son or daughter. When Musa asks God, however, he receives the answer that God has made her one of those who cannot conceive. Musa tells the woman about this response. When some time has passed, Musa sees this same woman carrying a small child. He approaches her and asks, ‘Is this your child?’ She confirms and explains that every time she was told that God had made her one of those who cannot conceive, she had uttered ‘*Ya Rahim*’, which addresses God as ‘the Merciful’ and is one of the names of Allah (Akkach, 2015). The repeated uttering of this name as a form of *duʿa* gave her hope, as she was convinced that God, being merciful, would provide for her. The speaker emphasised that despite her situation, she kept turning to God by using one of his names and kept her conviction that God would provide for her. Eventually, she was proved right.

The second narrative has at its centre a prominent medical doctor whose flight during an international journey is forced to make an emergency landing. The doctor, frustrated and eager to reach his destination, rents a car. While driving, he stops to pray and knocks on a random door. A woman opens, and inside the doctor sees a sick child. The woman says that the child is ill and that the only doctor able to save the child had no time to see them. However, she has made *duʿa* asking that this particular doctor will see the child. As it turns out, this is the precise doctor who, as if by accident, has knocked on their door. According to the speaker, the doctor broke down and cried when he realised that God had guided him to this child as a response to the woman’s prayer.

Both of these narratives emphasise the importance of asking God for help regarding material, concrete things—although not materialist or selfish desires. The two narratives’ respective resolutions likewise reinforce the power and willingness of God to respond in material, concrete ways. The stories thereby point to other effects of *duʿa* than increasing one’s spiritual level of proximity to God or developing an attitude of dependence on and trust in God, both of which have been emphasised by Islamic scholars (Katz, 2013; Khalil, 2011; Stjernholm, 2021). At the same time, the speaker points out that God might not respond in the way we had imagined or hoped for, since his knowledge about what we really need is greater than our own. Taken together, the two lectures presented here reinforce what is indicated in Fig. 1: the need to live within a *taqwa*-infused faith frame, constantly aware of God, and knowing that God will respond to sincere, repeated prayers.

The specific faith frame of a religious group is learned through a process, for example, through engaging with lectures promoting certain ontological commitments like those above. However, sometimes individuals express that religious teachings, when learned, articulate what they were already aware of; that they reflect their pre-reflexive certainty about the world and their own constitution. The 26-year-old Sana, who was engaged as a student and teacher in a Sufi mosque, said that in this mosque she had found what she had always felt to be true. She perceived herself to have been guided to this particular mosque and its shaykh. Sana had spent her teenage years searching for authentic Islamic knowledge in various mosques and Islamic institutes. The Sufi shaykh and community had taught her to ‘put into words’ the love and the values she had always carried with her. It just ‘made sense’, she explained (Interview, July 2019).

Specific faith frames are not necessarily known known by the individual prior to the experiences and feelings they are said to name. There can be various degrees of ‘fit’ between individual feelings and experiences on the one hand, and a faith frame on the other hand. For it to ‘make sense’, the perceived fit must be satisfying. At the same time, faith frames shape processes of sense-making. They affect the kinds of feelings and experiences one is likely to have, and they regulate which are perceived as religiously legitimate and important. Ultimately, our interlocutors frequently viewed this dynamic as being the work of God. Sana, as mentioned, felt guided by something outside of her to the Sufi mosque and its shaykh. Preachers, teachers, and students we encountered regularly explained events, like adopting a specific faith frame, with reference not only to the intensions or actions of human individuals or groups. Rather, from the point of view of the Danish Muslims we have listened to, faith frames ‘come to’ (Mittermaier, 2012: 248) the believer because their hearts and minds, being created by God, long for them.

### Cultivating nearness to God

To really have *taqwa*, rather than simply knowing that you *should* be constantly aware of God, demands continuous commitment to practices that nurture and maintain this awareness (Luhrmann, 2020: 21). In our material, we find an emphasis on cultivating the self to develop a constant state of *taqwa*. In this section, we analyse how Danish Muslims describe and reflect on ways to cultivate a sense of nearness to God—as well as the relevance of such processes of cultivation and purification. Many of Lyngsøe’s interlocutors expressed that their main motivation for engaging with Islamic knowledge was a desire to establish and maintain a close connection to God in their everyday life. The 24-year-old university student Sara, for example, said that her engagement with Islamic knowledge was a way to cultivate an awareness of the otherworldly and develop perceptiveness beyond the ordinary and everyday-like. When asked what motivated her to teach Islamic subjects, she replied:

I think it’s an important knowledge to have. So that everything doesn’t become so profane (*verdsligt*). Everyone would be nourished from engaging in and being aware of something that is not so profane. There should be a balance with also looking inwards, [acknowledging] that there exists something greater than yourself. Being conscious about that also makes you more humble (*ydmyg*) in your everyday life. (Interview, May 2019)

For Sara, engagement with Islamic knowledge was a way of connecting to spiritual aspects of life, thus committing to a faith frame and maintaining its relevance. The humbleness mentioned by Sara fits well within the ‘pious fear’ implied by *taqwa*. It implies a specific ontological status of the individual in a web of relations with an almighty God. Understanding one’s true constitution and relationship to the divine was a commonly expressed motivation for engaging with Islamic knowledge. This understanding would then establish a foundation for approaching God. The abovementioned Sana explained:

Everything is much easier to deal with (*kapere*) because you understand your relationship to God; God is made much more real (*virkeliggjort*) when you also understand the consequences of your actions, the positive consequences, that if you do like this, there is Paradise. (Interview, July 2019)

Continuously engaging with Islamic knowledge was described as a way to remind oneself of one’s relationship to God and to recommit to this connection in everyday life. Some women described how being knowledgeable was not in itself the main goal. Rather, engaging in a search for knowledge should maintain and improve one’s relationship to God by constantly reaffirming one’s *taqwa*. Seeking Islamic knowledge was a never-ending process in which there was always more to be learned; one continuously needed to reaffirm, remind, and recommit oneself in order to maintain and increase one’s *taqwa* and nearness to God, as Fig. 1 also indicates (cf. Gade, 2004; Lyngsøe, 2018).

The Qurʾan, in particular, was described as the ultimate, inexhaustible source for this process. In her study of Iranian women’s prayer practices, Niloofar Haeri (2021; see also 2017) describes how the Qurʾanic text invoked in prayer acquires multiple meanings depending on context, though the words remain the same. As the meanings of Qurʾanic passages are produced in an interaction between the text and the individual reciter, they develop over time in accordance with the memories, expectations, and emotions of the praying subject (Haeri, 2021: 90). We have encountered similar attitudes and narratives towards the meaning and power of Qurʾanic words. They were seen as universally meaningful, in the sense of containing wisdom for every situation. At the same time, a certain life situation can lead to the disclosure of a previously unknown meaning of a verse, thus giving the impression that the Qurʾan—meaning, ultimately, God—‘speaks’ to each specific moment of one’s own life.

In a semi-private women’s study group on interpretation of the Qurʾan (*tafsir*) led by Zainab, a woman in her 60s, this notion of the Qurʾan was fundamental to the participants’ engagement with the text. During classes, Zainab often emphasised that the participating women’s comments made her discover new aspects of verses despite having read them many times before (cf. Gade, 2004, 49; Haeri, 2021: 87–88). For one specific class during Ramadan 2020, the women had read the 99th sura, *al-Zalzala* (The Earthquake). After discussions on the meaning and benefits of its verses, Zainab suggested that the participants should integrate it into their evening prayer. She explained that after having read and discussed it during the day, it would attain a fresh meaning for everyone, as it would touch their hearts in a new way. Engaging closely with the words of the sura was thus perceived as both a way of opening the text for enhanced understanding and opening one’s heart to experiencing the effect of God’s words.

Prayer, in its different forms, was also highlighted as an especially potent time for developing nearness to God. In May 2019, Sana gave a lecture on the topic of achieving God’s friendship. Sana used both the Danish words for friend (*ven*) and friendship (*venskab*) as well as the Arabic term *wali* (plur. *awliya*), which she explained to be common in the Qurʾan. Within Sufi traditions, *awliya Allah* commonly refers to Sufi shaykhs or saints and implies that these possess extraordinary capacities due to their relationship to God (Ernst, 1997: 58–59; Radtke et al., 2012). When Sana talked about achieving God’s friendship, she did not equate her listeners with Sufi masters. Rather, she presented their intimate relationship to God as an ideal for every Muslim to strive for. Becoming God’s friend was, in this perspective, the very core of what it meant to be Muslim. To have God as one’s closest friend, Sana explained, meant to follow God in love and to trust that he will solve your problems and protect you from harm. She mentioned prayer as an important way to establish and affirm one’s relationship to God. Addressing God in prayer and remembering him in *dhikr* were ways of reminding oneself that ultimately one belongs to God, as well as acknowledging that God is present in each of our lives. Sana described prayer as a moment to establish a ‘direct connection’ to God (Field notes, May 2019). Prayer was thereby described as a repeated, explicit affirmation of a *taqwa*-infused faith frame, through which the praying subject performs a certain ontological commitment by acknowledging God’s existence (by addressing him), his agency (by asking for his intervention), and humans’ dependency on him (see also Mittermaier, 2019: 7; Bandak & Henkel, 2021).

Apart from being encouraged in Islamic educational settings like those described above, Muslim efforts to cultivate a close relationship to God are sometimes emphasised in forms of communication aimed at a broader public. One example is an episode of a Danish podcast series launched in 2021 called The Sharia Manifest (*Sharia Manifestet*). This podcast represents an in-depth thematic explication of a 43-point ‘Danish Muslim Manifest’ written by the two podcast hosts: Kasper Mathiesen, who has an MA in Islamic Studies and is a convert to Islam, and Naveed Baig, who has worked as hospital imam and is a PhD Candidate in Islamic theology at the University of Oslo.^8^ In an episode on the topic ‘Islam and Reformation’ (Islam og reformation 2020), the two hosts expanded on what they considered to be the most important form of Islamic reformation, namely that of the heart and one’s inner self. Here is an abridged excerpt of their conversation:

Kasper: The purification of the heart is a core tradition in Islamic history, in Islamic spirituality, the idea that humans can improve (*forbedre*) themselves. That our heart can be purified, the whole *tazkiyat al-nafs*,^9^ that is the ennobling (*forædling*) of the ego […], that you can get closer to Allah, that you can develop, that is the very core of Islam. […] Behind [the exterior] there is a much more important focus that is the relationship to God, what is called *muraqabat al-nafs*,^10^ meaning that you self-critically keep an eye on yourself, watch yourself, regulate yourself, in every little movement of the heart.

Naveed: Yes, so what we really mean with purification of the heart is talking to God. [...] Real reform is when we turn to God and God receives us and hears our prayers, [and gives] us his grace and his forgiveness and his mercy, and not least his love – that is what Muslims are after, God’s love [...]. In Islam we don’t need an intermediary (*mellemled*) to reach God, the basic Islamic teaching is precisely a reformation that says that the human becomes liberated and has access to God anywhere, at any time, and can pray to God whenever and wherever. So, there is already implicit in Islamic theology a reform movement in that humans (*mennesket*) should move towards God, and reach nearness (*nærhed*) and intimacy (*nærvær*) to God. (‘Islam og reformation’ 2020)

The language employed here is partly, though not exclusively, drawn from Sufi Islamic traditions. It should be noted that the rationale behind the supposed effectiveness of practices of purification, self-regulation, and prayer is an expectation that God will thereby be felt to be closer—this is why we have put ‘nearness to God’ at the centre of Fig. 1. As noted, there is an assumed reciprocal agency involved in this process. The pious subject acts in order to seek closeness to God, for example, through prayer, studies, and self-improvement. This is thought to open up for sensing an intimate nearness to and presence of God. In turn, God is expected to respond by coming near to, acting upon, and otherwise bless the pious subject. This sought-after reciprocity demands certain actions on the part of the believer, while the *taqwa*-infused faith frame simultaneously assumes certain actions on behalf of God.

### Experiencing nearness to God

When interviewed, Sana explained what it meant for her to feel connected to God and sense his presence in her life:

This light makes you more grounded *[rodfæstet]* in yourself; you do not hesitate (*flakker*) between choices in your life, you do not become as affected by other people’s opinions, but are more concerned with God’s thoughts about you *[tanker om én]*. I wish everyone could experience this, it is very beautiful. […] I can become fascinated by this myself, shed tears over how lucky I am to be carried through life in this way. (Interview, July 2019)

For Sana, God’s nearness was experienced in different ways, although here she described it as ‘light’.^11^ Basically, she sensed it as an ever-present guidance, keeping her on track and affirming that ultimately, her existence depended on God. For Sana, who was associated with a Sufi tradition, the cultivation of *ikhlas*, ‘a unity and purity of [the] interior gaze which is directed at God and God alone’ (Gardet, 2012), made room for God’s light to shine within her. This affected her mode of being in the world, her ontological commitment. She talked about being ‘carried’ rather than ‘going’ through life, indicating an agency other than her own. Sana said that she felt supported by God through the joys and difficulties of life. This helped her to orient herself towards the will of God rather than the opinions of her acquaintances. She described the feeling of God’s nearness as a bodily sensation of ease, calmness, and meaningfulness. Sana also felt that God equipped her with more time than other people, for example, her non-Muslim colleagues. She said:

Some people ask me, ‘how do you find the time for all the things you do?’ But I don’t feel stressed, and I believe it is the spiritual aspect (*det spirituelle*) that magically provides me with more hours in the week. The spiritual gives every day a specific blessing. Of course, everything is not all rosy (*lyserødt*), some periods are more stressed than others, but it is not common. (Interview, July 2019)

Sana sensed God’s impact on her everyday life as a qualitative alteration of time—not in a metaphorical sense, but concretely. She also gave examples of people from her mosque who similarly received help from God in the form of needed extra time or power.

Often, interlocutors voiced implications of God’s nearness on their life as an expectation, something that they longed for or hoped would happen. This typically related to active pious cultivation of the relationship to God, for example, through prayer. The actual experience of God’s nearness and agency, however, was said not to be caused by human actions. Cultivation was rather a process of opening oneself up to the active work of God (Luhrmann, 2020; Mittermaier, 2011, 2012). Again, there is a perceived reciprocal agency where the individual’s intentional work can be supplemented by sensing God as ‘an active player’ in the world (Mittermaier, 2019, 8). When Louise, who was introduced in the article’s beginning, was asked what had led her to join the specific Sufi mosque she frequented, she explained that she had been guided by God’s intervention. She recognised that from an outsider’s point of view, it might seem coincidental. However, to Louise it was not. She narrated a series of events relating to her conversion that she *knew* resulted from God’s guidance. She had taken an interest in Islam as a young adult and gradually came to seek more advanced Islamic learning. Regarding this seeking, she said: ‘It’s my perception that I received help from God to have this placed within me’ (Interview, May 2019). During a time of loneliness after turning her back on what she called unhealthy, un-Islamic relationships, she came upon an old friend from high school who was Muslim—‘coincidentally, in quotation marks’ (Interview, May 2019). It was also through God’s intervention, Louise said, that she found herself living next door to the shaykh whom she would later come to consider her spiritual guide. Summing up, Louise said:

I have constantly been given (*givet*) what is right. I could easily have found something related to Salafi teachings, but in my heart, I could just sense that that interpretation of Islam is not right. (Interview, May 2019)

The point here is that Louise perceived this sensation in her heart as resulting from God’s guidance. Just like she was convinced that God had guided concrete events, her feelings were similarly affected by God. From Louise’s and Sana’s narratives, it is clear that the experience of God’s nearness also informs and affirms their faith frame as well as the semiotic ideology through which they perceive the world (see Fig. 1).

Both Sana and Louise experienced the nearness of God on a mental level, affecting their feelings, moods, and abilities. Yet they also reported sensing God’s impact more concretely, as guidance through and causation of actual events. For Louise, an example of the latter was that she unknowingly came to live next door to the person who would become her shaykh. Louise self-consciously reflected on her own story, and the fact that it deviated from common perceptions of human agency and intentionality (Groeninck, 2017: 179f; see also Barad, 2007). ‘It’s hard to explain to a non-religious person’, she said, but elaborated:

It really is as if it is not a decision you take from your own cognitive, reflected process. It is as if your heart takes the decision for you. That’s also how it was in [the mosque]. I just knew I was in the right place. It was an altogether different feeling. (Interview, May 2019)

As described by Sana and Louise, being in the world as a Muslim is to experience God’s nearness both in one’s interior and as signs to interpret in the exterior world (cf. Groeninck, 2017: 167f). Experiencing and acknowledging the work of God within oneself and in one’s life, as in Louise’s refusal to see events as coincidental, is an affirmation of an Islamic faith frame that is promoted, for example, by local Islamic scholars, teachers, and preachers like those cited above.

## Concluding discussion

In our analyses, we have pointed to the central importance ascribed to learning about, cultivating, and experiencing nearness to God in our material. In the first section, we presented examples of how a focus on *taqwa* was used in a mosque-affiliated lecture to encourage the audience to strive for a constant awareness of God. In a lecture on *duʿa*, the speaker emphasised the benefits and concrete effects that approaching God directly through prayer can have.

The next section pointed to the process of cultivating individual nearness to God. As shown, crucial ways of doing so are by engaging with Islamic learning and approaching God in prayer. Rather than being mainly done out of a sense of obligation, we find that our interlocutors emphasise the benefits and rewards they get out of such practices because they further a nearness to God. Engaging in learning and prayer are ways of actively opening oneself up to sensing God’s omnipresence and nearness, thus committing to a specific faith frame that includes a perceived reciprocity in the relationship between God and the individual Muslim. Individuals’ spiritual openness and sincerity can be cultivated through practices of learning and prayer, ostensibly bringing the individual closer to God.

In the last section, we discussed the narratives of two women who experienced that God ‘carries’ them through life, ‘gives’ them what is right, and ‘places’ knowledge ‘within their hearts’, so that life is lived through God’s active guidance. Both women acknowledged that their experiences were not likely to be taken at face value by a non-religious person. However, Sana’s and Louise’s accounts did not include particularly dramatic or miraculous events. Rather, their stories point to a sense of intimacy with God that is sensed in the mundane realities of everyday life (cf. Schielke, 2019). They represent an ontological commitment that includes the comforting and guiding presence of God; or, as Luhrmann (2020: 21) phrases it, ‘a sustained, intentional, deliberative commitment to the idea that invisible beings are involved in human lives in helpful ways’.^12^

How can we understand the interrelatedness between the ideas, practices, and experiences concerning Danish Muslims’ nearness to God that we have presented in this article? We have proposed a model (see Fig. 1) that illustrates how a sense of nearness to God can become established and maintained. This included the notion of a *taqwa*-infused faith frame, building on Luhrmann’s vocabulary. This faith frame represents a specific ontological commitment with certain rights and wrongs, which the individual typically learns about through various types of religious instruction. An example from our analysis is the conviction that God *will* respond to those who make sincere *duʿa* to him. We have further combined this with Keane’s concept semiotic ideology in order to capture that people interpret signs—such as words, movements, bodily sensations, and actions—differently. A particular faith frame, in this case one that we have called *taqwa*-infused, will support a certain semiotic ideology, according to which individuals who successfully inhabit the faith frame are likely to interpret various signs. An example from above is the sense that God speaks directly to your own life experience when you recite the Qurʾan—rather than it simply being a regular text. The interpretation of signs according to a certain Islamic semiotic ideology furthermore opens up for possible experiences of divine intervention and active guidance, as pointed out by Mittermaier and others. Occurrences that may seem perfectly ordinary to a non-religious individual may be interpreted as clear signs of divine intervention or guidance by a person inhabiting a *taqwa*-infused faith frame. As an example, we can recall Louise’s refusal above to accept certain events in her life as being coincidental. We have further suggested that the faith frame includes an expectation—which can support experiences—of a reciprocal agency, whereby the individual’s efforts to draw near to God is felt to be ‘answered’ by God, who in turn draws nearer to the individual. Such experiences are likely to feed into an increased conviction of the truthfulness and usefulness of the *taqwa*-infused faith frame, meaning that the three dynamics we have pointed to in Fig. 1 are interrelated in a mutually reinforcing way.

To conclude, we are pointing to an aspect of Muslim piety where *taqwa*-infused faith frames allow individual Muslims to pursue and experience a reciprocal nearness to God as a comforting and guiding agency in their lives. We suggest that more attention should be paid to the work done by Muslims in different cultural environments in order to draw near to God as well as to the effects this is alleged to have. In this article, we have attempted to take such a mode of analysis one step further by pointing out three interrelated yet distinct dynamics of how a felt nearness to God can come about.

## Data Availability

Not applicable.

## Footnotes

1 Although we limit ourselves to introducing the concept of *taqwa* here, there are several related concepts that would make good sense to include in an extended treatment. For example, both *ikhlas* (sincerity) (Gardet, 2012) and *niyya* (intention) (Wensinck, 2012), whose uses and meanings are related to that of *taqwa*, are regularly used in the environments from which our data have been gathered.

2 Luhrmann also integrates references to Islamic prayer, especially through the works of Gregory Simon (2009) and Niloofar Haeri (2021; see also Haeri, 2013, 2017). These are sources of inspiration to us as well.

3 The video was published by the YouTube channel “Muslimsk Ungdom i Danmark” (Muslim Youth in Denmark, abbreviated MUNIDA) and was last accessed at https://youtu.be/ZUQp9qSlnuE on 28 March 2022, when it had 200 views. Despite the lecture’s limited number of views, it is useful here as an example of a *type* of Islamic teaching that recurrently takes place in this Islamic environment, rather than a singular piece of oratory that is particularly interesting due to its uniqueness. The speaker does not mention from where the hadith is taken, but it can be found in Nawawi’s collection of 40, taken from Tirmidhi.

4 The quotation was first made in Arabic, *ataqillah haithuma kunta*, and then translated into Danish. All translations from Danish were done by the authors.

5 Here, we have translated the oral rendering in Danish by the speaker. In Abdel Haleem’s translation, this section of 49:13 reads: ‘In God’s eyes, the most honoured of you are *the ones most mindful of Him*’ (our italics). The Arabic term here is *atqakum*, i.e. the ones who have more *taqwa*. In 10:62, the people specified are the *awliya Allah*, which is often translated as ‘friends of God’ – Abdel Haleem renders it ‘those who are on God’s side’ – and used about spiritual Sufi masters. The notion of being ‘God’s friend’ is further mentioned by an interlocutor below. The following verse, 10:63, speaks of the *yattaqun*, the ones with *taqwa*.

6 The video was published by the YouTube channel “Muslimsk Ungdom i Danmark” (Muslim Youth in Denmark, abbreviated MUNIDA) and was last accessed at https://youtu.be/M220rXDyWKs on 28 March 2022, when it had 311 views. Regarding the relevance of analyzing a video with a limited number of views, see comment in footnote 4.

7 In Abdel Haleem’s rendering: ‘Call on Me and I will answer you’.

8 More information (in Danish) about the manifest and the podcast can be found at https://www.danskmuslimskmanifest.dk/ (last accessed 28 March 2022). The manifest and the podcast have both received a fair amount of attention in Danish print and broadcast media.

9 The Islamic (especially Sufi) concept of *tazkiyat al-nafs*, or ‘purification of the self’, has been dealt with thoroughly by Gavin Picken (2005, 2011).

10 This concept within Islamic spirituality refers to a constant vigilant and watchful surveillance of one’s inner self, in order to keep one’s spiritual focus on God alone rather than egoistic desires. For further explanation and context, see e.g. the multiple occurrences of *muraqaba* in Chiabotti et al. (2017).

11 There is a long and rich Islamic tradition of associating ‘light’ (*nur*) with God (Lange, 2021) as well as with the prophet Muhammad (Andani, 2020).

12 Although we have focused on positive experiences of nearness to God (following our material), experiencing oneself to be entangled in webs of relationships to unseen actors—such as gods, spirits, and demons—can certainly also be negative, including feelings of fear, anxiety, and lack of personal freedom. Although we have not been able to engage thoroughly with the existing literature on Islamic exorcism and jinn possession here, it would be interesting to study the ‘helpful’ and the ‘harmful’ webs of relationships alongside each other.

## References

[CR1] Akkach S, Fleet K, Krämer G, Matringe D, Nawas J, Rowson E (2015). Beautiful names of Allah. Encyclopaedia of Islam, THREE.

[CR2] Andani K (2020). Metaphysics of Muhammad: The Nur Muhammad from Imam Jaʿfar Al-Sadiq (d. 148/765) to Nasir Al-Din Al-Tusi (D. 672/1274). Journal of Sufi Studies.

[CR3] Andersen PB, Erkmen J, Gundelach P, Frederiksen M (2019). Udviklingen i (ikke)religiøsitet. Usikker modernitet. Danskernes værdier fra 1981 til 2017.

[CR4] Asad T (1993). Genealogies of religion. Discipline and reasons of power in Christianity and Islam.

[CR5] Bangstad, S. (2014). *The politics of mediated presence: Exploring the new Muslim voices in the contemporary mediated public spheres in Norway*. Spartacus.

[CR6] Bandak A, Henkel H (2021). Bøn: Antropologiske åbninger i arbejdet med religiøs praksis. Tidsskriftet Antropologi.

[CR7] Barad K (2007). Meeting the universe halfway.

[CR8] Bialecki J (2014). Does God exist in methodological atheism? On Tanya Luhrmann’s when God talks back and Bruno Latour. Anthropology of Consciousness.

[CR9] Bialecki J (2016). Diagramming the will: Ethics and prayer, text, and politics. Ethnos.

[CR10] Chiabotti F, Feuillebois-Pierunek E, Mayeur-Jaouen C, Patrizi L (2017). Ethics and Spirituality in Islam.

[CR11] Ernst CW (1997). Sufism: An essential introduction to the philosophy and practice of the mystical tradition of Islam.

[CR12] Fadil N (2019). The Anthropology of Islam in Europe. A double epistemological impasse. Annual Review of Anthropology.

[CR13] Furani K, Robbins J (2021). Introduction: Anthropology within and without the secular condition. Religion.

[CR14] Gade AM (2004). Perfection makes practice: Learning, emotion, and the recited Qur’an in Indonesia.

[CR15] Groeninck, M. (2017). Reforming the self, unveiling the world. Islamic religious knowledge transmission for women in Brussels’ mosques and institutes from a Moroccan background. PhD dissertation, KU Leuven.

[CR16] Gardet, L. (2012). Ik̲h̲lāṣ. In: P. Bearman, TH. Bhianquis, C. E. Bosworth, E. van Donzel, & W. P. Heinrichs (Eds.), *Encyclopaedia of Islam, Second Edition*. Brill. 10.1163/1573-3912_islam_SIM_3512

[CR17] Guhin J (2019). Defining Duʿā’: A study of contested meanings in immigrant Muslim schools in the New York City area. The Journal of Education in Muslim Societies.

[CR18] Haeri, N. (2013). The private performance of salat prayers: Repetition, time, and meaning. *Antropological Quarterly 86*(1), 5–34. 10.1353/anq.2013.0005

[CR19] Haeri N (2017). The sincere subject. Mediation and interiority among a group of Muslim women in Iran. HAU: Journal of Ethnographic Theory.

[CR20] Haeri N (2021). Say what your longing heart desires: Women, prayer and poetry in Iran.

[CR21] Hirschkind, C. (2006). *The ethical soundscape: Cassette sermons and Islamic counterpublics*. Columbia University Press.

[CR22] Islam og reformation. (2020). *Shariamanifestet* (The Sharia Manifest). Podcast episode, 10 October 2021. Retreived March 28, 2022, from https://www.danskmuslimskmanifest.dk/podcast-sharia-manifestet/episode/4b34e595/islam-og-reformation

[CR23] Jensen PF (2019). Om moskéer og medborgerskab. Moskéers rolle for aktivt medborgerskab som oplevet af muslimske kvinder i Danmark. Tidsskrift for Islamforskning.

[CR24] Katz MH (2013). Prayer in Islamic thought and practice. Cambridge University Press.

[CR25] Keane W (2003). Semiotics and the social analysis of material things. Language and Communication.

[CR26] Keane, W. (2007). *Christian moderns: Freedom and fetish in the mission encounter*. University of California Press.

[CR27] Keane W (2018). On semiotic ideology. Signs and Society.

[CR28] Khalil A (2011). Is God obliged to answer prayers of petition (*Duʿa*)? The Response of classical Sufis and Qurʾanic exegetes. Journal of Medieval Religious Cultures.

[CR29] Lange C (2021). Eternal sunshine of the spotless mind: Light and luminous being in Islamic theology. Critical Research on Religion.

[CR30] Lewisohn, L. (2012). Taḳwā. In P. Bearman, Th. Bhianquis, C. E. Bosworth, E. van Donzel, & W. P. Heinrichs (Eds.), *Encyclopaedia of Islam, Second Edition*. Brill. 10.1163/1573-3912_islam_COM_1457.

[CR31] Liebman LL, Galal L (2020). Classing religion, resourcing women. Muslim women negotiating space for action. Cultural Dynamics.

[CR32] Luhrmann, T. M. (2007). How do you learn to know that it is God who speaks? In D. Berlin & R. Sarró (Eds.), *Learning Religion: Anthropological Approaches* (83–102). Berghahn Books. https://www.jstor.org/stable/j.ctt9qcmz4.9. Accessed 25 Oct 2022

[CR33] Luhrmann, T. M. (2012). *When God talks back: Understanding the American evangelical relationship with God*. Vintage.

[CR34] Luhrmann, T. M. (2020). *How God becomes real: Kindling the presence of invisible others*. Princeton University Press.

[CR35] Lundby, K. (2018). Contesting religion: The media dynamics of cultural conflicts in Scandinavia. *De Gruyter*. 10.1515/9783110502060

[CR36] Lyngsøe M (2018). Koranen er lyd. Koranrecitation som den læres bruges og forstås af kvindelige københavnske muslimer. Tidsskrift for Islamforskning.

[CR37] Mahmood S (2012). Politics of piety. The Islamic revival and the feminist subject.

[CR38] Mittermaier, A. (2011). *Dreams that matter: Egyptian landscapes of the imagination*. University of California Press.

[CR39] Mittermaier A (2012). Dreams from elsewhere: Muslim subjectivities beyond the trope of self-cultivation. Journal of the Royal Anthropological Institute.

[CR40] Mittermaier, A. (2019). *Giving to God. Islamic Charity in Revolutionary Times*. University of California Press.

[CR41] Mittermaier A (2021). Beyond the human horizon. Religion & Society.

[CR42] Ohlander ES (2005). Fear of God (*taqwā*) in the Qurʾān: Some notes on semantic shift and thematic context. Journal of Semitic Studies.

[CR43] Orsi RA, Orsi RA (2012). The problem of the holy. The Cambridge Companion to Religious Studies.

[CR44] Orsi RA (2016). History and presence.

[CR45] Otterbeck, J. (2010). *Samtidsislam: Unga muslimer i Malmö och Köpenhamn*. Carlsson.

[CR46] Picken G (2005). Tazkiyat al-nafs: The Qur’anic Paradigm. Journal of Qur’anic Studies.

[CR47] Picken, G. (2011). *Spiritual purification in Islam: The life and works of al-Muhasibi*. Routledge.

[CR48] Poulsen, J. A., K. Trolle, M. F. Larsen and B. S. Mortensen. (2021). *Religiøsitet og forholdet til folkekirken 2020. Bog 1: Kvantititave studier.* Folkekirkens Uddannelses- og Videnscenter.

[CR49] Radtke, B., Lory, P. Zarcone, T. H., DeWeese, D., Gaborieau, M., Denny, F. M., Françoise Aubin, Hunwick, J. O., & Mchugh, N. (2012). Walī. In P. Bearman, Th. Bhianquis, C. E. Bosworth, E. van Donzel, & W. P. Heinrichs (Eds.), *Encyclopaedia of Islam, Second Edition*. Brill. 10.1163/1573-3912_islam_COM_1335.

[CR50] Robbins J (2001). God is nothing but talk: Modernity, language, and prayer in a Papua New Guinea society. American Anthropologist.

[CR51] Robbins J (2017). Keeping God’s distance: Sacrifice, possession, and the problem of religious mediation. American Ethnologist.

[CR52] Robbins J (2019). On knowing faith: Theology, everyday religion, and anthropological theory. Religion and Society.

[CR53] Rytter M (2016). By the beard of the prophet. Imitation, reflection and world transformation among Sufis in Denmark. Ethnography.

[CR54] Rytter M (2019). Writing against integration: Danish imaginaries of culture, race and belonging. Ethnos.

[CR55] Rytter M (2019). The hair of the Prophet. Relics and the affective presence of the absent beloved among Sufis in Denmark. Contemporary Islam.

[CR56] Schielke, S. (2019). The power of God: Four proposals for an anthropological engagement. *ZMO Programmatic Texts* 13. Retrieved October 25, 2022, from https://www.ssoar.info/ssoar/handle/document/61209

[CR57] Simon G (2008). The soul freed of cares. Islamic prayer, subjectivity, and the contradictions of moral selfhood in Minangkabau, Indonesia.&nbsp;. American Ethnologist.

[CR58] Stjernholm S (2021). Närmre Allah: Muslimska predikanter om privat *duʿa*. Tidsskriftet Antropologi.

[CR59] Suhr C (2019). Descending with Angels.

[CR60] *The Qurʾan: A New Translation by M. A. S. Abdel Haleem* (2004). Oxford University Press.

[CR61] Thurfjell D, Willander E (2021). Muslims by ascription: On post-Lutheran secularity and Muslim immigrants. Numen.

[CR62] Wensinck, A. J. (2012). “Niyya.” In P. Bearman, Th. Bianquis, C. E. Bosworth, E. van Donzel, & W. P. Heinrichs (Eds.), *Encyclopaedia of Islam, Second Edition*. Brill. 10.1163/1573-3912_islam_SIM_5935

